# Children’s Body composition and Stress – the ChiBS study: aims, design, methods, population and participation characteristics

**DOI:** 10.1186/0778-7367-70-17

**Published:** 2012-08-09

**Authors:** Nathalie Michels, Barbara Vanaelst, Krishna Vyncke, Isabelle Sioen, Inge Huybrechts, Tineke De Vriendt, Stefaan De Henauw

**Affiliations:** 1 Department of Public Health, Faculty of Medicine and Health Sciences, Ghent University, De Pintelaan 185, 2 Blok A, B-9000 Ghent, Belgium; 2 Research Foundation – Flanders, Egmontstraat 5, B-1000 Brussels, Belgium; 3 Department of Health Sciences, Vesalius, Hogeschool Gent, Keramiekstraat 80, B-9000, Ghent, Belgium

**Keywords:** Stress, Child, Body composition, Obesity, Cortisol, Heart rate variability, Questionnaire, Food habits, Physical activity, Sleep

## Abstract

**Background:**

The last decades, the prevalence of childhood obesity has increased. Apart from other lifestyle factors, the effect of chronic psychosocial stress on the development of obesity has been recognized. However, more research is needed into the influence of chronic stress on appetite regulation, energy balance and body composition, as well as on the interaction with physical activity/sedentary behavior, diet and sleep in children. In this regard, the ChiBS study (Children’s Body composition and Stress) was designed at the Ghent University. Within this paper, we describe the aims, design, methods, participation and population characteristics of the ChiBS study.

**Methods:**

The influence of chronic stress on changes in body composition is investigated over a two-year follow-up period (February-June 2010, 2011 and 2012) in primary-school children between 6 and 12 years old in the city Aalter (Flanders, Belgium).

Stress is measured by child- and parent-reported stress-questionnaires, as well as by objective stress biomarkers (serum, salivary and hair cortisol) and heart rate variability. Body composition is evaluated using basic anthropometric measurements and air displacement plethysmography. Additional information on socio-economic status, medical history, physical activity, dietary intake and sleep are obtained by questionnaires, and physical activity by accelerometers.

**Results:**

The participation percentage was 68.7% (N = 523/761), with 71.3% of the children willing to participate in the first follow-up survey. Drop-out proportions were highest for serum sampling (12.1%), salivary sampling (8.3%) and heart rate variability measurements (7.4%).

**Discussion:**

The ChiBS project is unique in its setting: its standardized and longitudinal approach provides valuable data and new insights into the relationship between stress and changes in body composition in a large cohort of young children. In addition, this study allows an in-depth investigation of the validity of the different methods that were used to assess stress levels in children.

## Background

The last decades have been characterized by a global growing obesity epidemic, starting already in childhood [1,2]. World-wide at least 110 million children are overweight or obese [2]. In the European Union, the prevalence of childhood overweight and obesity ranges from 10-20% (northern European areas) to 20-40% (Mediterranean Sea countries) and is expected to rise by 1.3 million children per year [3]. These numbers stress the importance of a better understanding of the complex etiology of obesity in order to help developing effective prevention programs. In addition, evidence confirmed the importance of focusing obesity prevention on young age groups, as obese children were found to be at increased risk of becoming obese adults (“tracking phenomenon”) [4] and to experience increased metabolic complications in adulthood [5]. To simplify, overweight and obesity will be covered by the term ‘obesity’ in this article.

Excessive caloric intake, insufficient physical activity and sleep deprivation are major lifestyle factors involved in the development of obesity [6]. Recently, the effects of chronic psychosocial stress have been increasingly recognized, also in children [7-10]. Chronic exposure to stress may disrupt the physiological stress system, influencing food intake regulation (increased energy intake and craving for ‘comfort foods’) [11,12] and fat deposition in the body (favoring central obesity) [9,11,13]. However, there is a need for more scientific research into the mechanisms linking chronic stress to appetite regulation, energy balance and consequently body composition in humans and more importantly in children.

**Figure 1:**
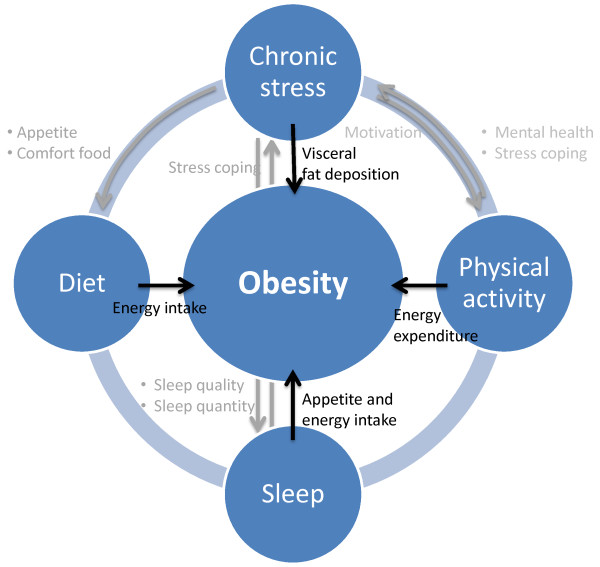
**Lifestyle factors involved in the development of obesity and investigated in the ChiBS project *Grey arrows indicate the study hypotheses, black arrows show the effect of the four lifestyle factors on obesity***.

The stress-obesity relationship is characterized by direct and indirect pathways (Figure 1). The direct effect of stress on body fatness and consequently the development of obesity is largely caused by the end-product of the hormonal stress response, i.e. cortisol [14]. Cortisol favors visceral fat disposition and stimulates appetite [9,11,13]. In addition, stress may indirectly facilitate the development of obesity by influencing other lifestyle factors such as diet, physical activity and sleep [8,9]. After all, stressed persons may consume more so-called “comfort foods”, as these foods stimulate rewarding and pleasure sensations [11,12]. Furthermore, stressed persons may be less motivated or have less energy to do physical activity and may suffer from sleeping problems [9]. Inversely, these lifestyle factors may also influence the stress load. Physical activity may be a protecting factor against obesity and stress by increasing energy expenditure and by improving mental health and stress coping [15]. On the contrary, lack of sleep may reduce coping capacity and thus resistance against stress [9,16].

The ChiBS study (Children’s Body composition and Stress) is designed to investigate the relationship between chronic psychosocial stress in young children (6-12 years old) and changes in body composition (body fat) over a two-year follow-up period (2010-2012). It is hypothesized that the exposure to chronic stressors may affect children’s body composition in the long-term by promoting body fatness increase and the development of obesity. More specifically, this study will examine the influence of chronic stress on the evolution of different body composition parameters longitudinally, taking into account diet, sleep and physical activity as intermediary factors in this relationship. It is hypothesized that chronic stress may promote consumption of energy dense highly palatable (sugar and fat rich) foods and a deviant eating behavior, decreased quantity and quality of sleep and a decreased amount of physical activity, as indicated in Figure 1. To accurately measure stress, child- and parent-reported stress questionnaires as well as objective stress biomarkers from different biological matrices are used. A second, parallel aim is to test the feasibility and interrelationships of these different stress measurements in children. Finally, the third aim is to further unravel the impact and mutual relationships of physical activity, diet, sleep and stress.

This paper describes the design of the ChiBS study, its instruments, measurements, population characteristics, and participation and drop-out rates for each examination module.

## Methods

### Study design and sampling

To study the relationship between chronic stress and changes in body composition over a two-year follow-up period, the ChiBS study was designed as a prospective cohort study. Approach and enrolment of the participants for the baseline survey of ChiBS (February 2010) was largely simplified by integrating it in the IDEFICS project (Identification and prevention of dietary- and lifestyle-induced health effects in children and infants; funded by the European Sixth Framework Programmed [17]). Indeed, the baseline survey of ChiBS coincided with the first follow-up survey (school-year 2009-2010) of the Belgian control cohort (i.e. in the city Aalter) of the IDEFICS study. All children participating in the control section of this IDEFICS survey (N = 761) were eligible to join the ChiBS study.

At baseline, the children were between 5 and 11 years old (last year of kindergarten and first four years of elementary school). Their parents were individually contacted by providing a letter via the schools, wherein they were informed and invited to let their children participate in the ChiBS project. Parents were asked to sign a consent form, in which the option was offered to participate in the full ChiBS programme or in a selected set of measurement modules. The children are being followed-up in a second and third survey module of the ChiBS study, to be conducted in February-June 2011 and February-June 2012 to fully cover primary-school age. For both follow-up surveys, the parents of participating children are contacted telephonically and are asked to sign a new consent form, in which options for all separate examination modules are offered. The fieldwork is conducted partly at school and partly at the municipal sports park of the city Aalter (permanent localization of materials e.g. BODPOD®). The ChiBS study is conducted according to the guidelines laid down in the Declaration of Helsinki and is approved by the Ethics Committee of the Ghent University Hospital.

**Figure 2:**
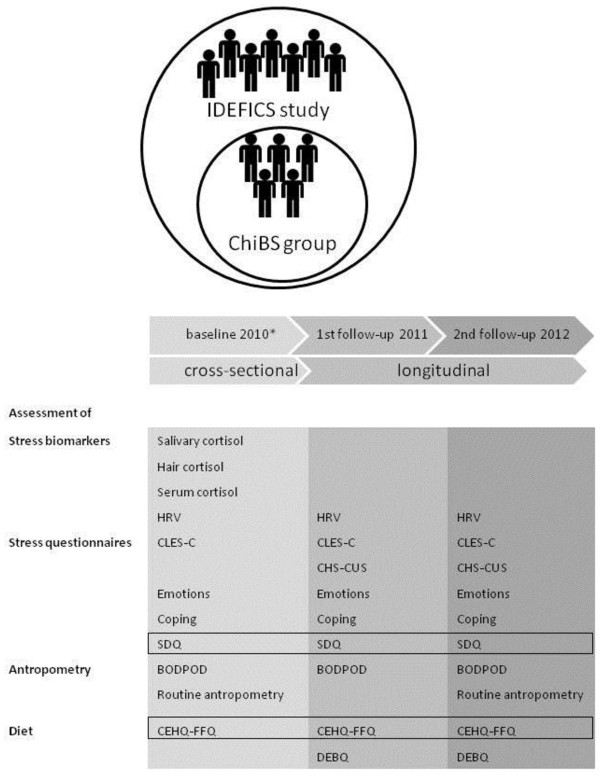
**Timeline of the ChiBS project and corresponding measurements of each survey period.** All measurements are conducted at the municipal sports park of Aalter, except for the salivary, hair and serum sampling and routine anthropometric measurements of the baseline ChiBS survey which were conducted at the schools. The questionnaires placed in a frame were administered to the parents, not to the children. *Administration of the stress questionnaires and bio-sampling at the baseline survey were restricted to elementary-school children (not children from kindergarten). In the first and second follow-up survey all children were of elementary-school age. CEHQ-FFQ = Children’s Eating Habits Questionnaire-Food Frequency Questionnaire, CHS-CUS = Children’s Daily Hassles and Daily Uplifts Scale, CLES = Coddington Life Events Scale, DEBQ = Dutch Eating Behaviour Questionnaire, HRV = heart rate variability, SDQ = Strengths and Difficulties Questionnaire.

Figure 2 schematically presents the timeline of the ChiBS project and the corresponding measurements and examinations of each survey period. While ChiBS’ examinations focus on stress-assessment (i.e. questionnaires and biomarkers), body-composition measurements and eating behavior; additional information on socio-demographics (e.g. parental education, income, place of birth), sleep, medical conditions, dietary intake and physical activity is obtained as part of the IDEFICS study, collected at the baseline ChiBS survey [17].

### Measurements and examinations

#### Stress biomarkers

In analyzing stress, the two most important stress-pathways are covered using heart rate variability (HRV) representing the autonomic nervous system [18,19] and cortisol measurements representing the hypothalamic-pituitary-adrenal stress system [14,20]. Cortisol is the most commonly used stress biomarker since both the normal pulsation and rhythmic fluctuation in cortisol production is sensitive to on-going life stressors [21]. Cortisol can be measured in different biological materials. Each type of biological material has its own strengths and limitations and carries partially different information as each tissue type reflects different cortisol levels from a different time frame (i.e. minutes to hours for singular serum and saliva samples, one day for urine and weeks to months for salivary series and hair) [20,22,23]. As such, three different biological samples are collected in the ChiBS study, including serum, salivary and hair samples, to cover the short and long-term stress exposure. Furthermore, these three different sample types allow an in-depth investigation of the validity, feasibility and intercorrelations of cortisol measurements in serum, saliva and hair among children.

##### Salivary cortisol

Saliva was collected into Salivette synthetic swabs especially designed for cortisol analysis (Sarstedt, Germany). The participating children were asked to collect saliva during two consecutive weekdays at four time points: immediately on awakening (T0), 30 minutes after waking-up (T30), 60 minutes after waking-up (T60) and in the evening between 7 and 8 PM (Tev). After all, cortisol secretion has a circadian rhythm with low levels in the evening/night and high levels in early morning and there is also a cortisol awakening response (CAR) showing a quick cortisol increase within 30 minutes after awakening. This collecting scheme allows to obtain information on both the circadian rhythm and the CAR [24]. To standardize sample collection, sampling and storage instructions were provided in a manual (see Additional file 1). The parents were also asked to fill in a checklist about instruction compliance (modified from Hanrahan et al. [25]): (1) awakening time, collection hours, health condition, physical activity, caffeine consumption and medication on the collection days and (2) the compliance to food restriction and teeth brushing one hour before the sampling. Analysis was done using an electrochemiluminescence immunoassay (ECLIA) [26]. Samples of corticosteroid-users, as well as morning samples collected more than 5 minutes too late/early and evening samples not collected between 7 PM and 9 PM were excluded. The detailed descriptive results were published elsewhere [27].

##### Hair cortisol

The ChiBS study functions additionally as a pilot project evaluating the feasibility and validity of hair cortisol (and cortisone) measurements in healthy children and its applicability for large-scale psychosocial stress research in young children, as this is currently unexplored. Cortisol (and cortisone) concentrations were analyzed in hair samples obtained from the vertex posterior region of the scalp [28,29]. Sampling was done at school by trained researchers. The hair samples were cut as close to the scalp as possible using clean, stainless steel scissors and tied together with a little cord to mark the proximal side. Only the most proximal 6 cm of the hair strands were analyzed (approximately 150 to 200 mg of hair), which is generally assumed to be the maximum length of hair to obtain a reliable estimate of systemic cortisol concentrations in the past (i.e. 6 months) [29]. Hair samples were exclusively taken from girls to maximize the possibility that the hair reached the required length. If the length of hair was shorter than 6 cm, no sample was taken. The hair samples were stored in a folded piece of paper in individual zip-lock bags in a dark, dry place and at constant temperature, to be analyzed 12 months later at the University of Strasbourg in the Institute of Legal Medicine (Faculty of Medicine). Hair cortisol has been shown to be stable over months to years, justifying the long time lag between sampling and analyzing. An amount of 50 mg of hair was finely minced, after which cortisol was extracted from the hair samples. Cortisol concentrations were analyzed using a Liquid Chromatography-tandem Mass-Spectrometry technique (Acquity UPLC Waters, Quattro Premier XE and Mass Lynx software). The details of the methods and the validation parameters are described in a separate paper.

##### Serum cortisol

After an overnight fasting period, blood samples were obtained through venipuncture using local anesthesia with EMLA® patches (lidocaine + Prilocaine). Blood samples were centrifuged and serum was stored at -80°C. Serum cortisol was assayed using the same technique as salivary cortisol namely ECLIA.

##### Heart rate variability

HRV is defined as the variability of the time between consecutive R peaks of the electric QRST interval on the electrocardiogram. In a healthy situation, considerable variability reflects the heart’s ability to adequately respond to physiological and environmental stimuli. It is hypothesized that chronic stress might facilitate a decreased HRV [30].

Each child was individually examined in a quiet room in supine position (i.e. lying down with the face up) during 10 minutes. Children were asked to refrain from strenuous physical activity on the measurement day. The child was encouraged to be calm, to breath normally and not to speak or move during the 10 minutes of HRV measurement. The heart rate belt was fixed around the chest and measurements were started after a couple of minutes when the signal was stabilized. RR-intervals (RRI) were recorded at a sampling rate of 1000 Hz with the elastic electrode belt Polar Wear link 31 using a Wind link infrared computer transmitter. This low-cost device has a proven validity compared to the gold standard of an electrocardiogram device [31], also in children [32]. Data processing to obtain time-domain and frequency-domain parameters was performed with the free, professional HRV Analysis Software of the University of Kuopio, Finland [33]. Low frequency (LF) and high frequency (HF) bands were analyzed between 0.04-0.15 Hz and 0.15-0.4 Hz, as default [30]. The RR series were de-trended using the Smoothness priors method [34] with alpha = 300 and a cubic interpolation at the default rate of 4 Hz was done. The middle 5 minutes were manually checked on their quality and if necessary, another appropriate 5 minutes interval was chosen. Quality was defined as no large RRI outliers, an equidistance between consecutive RRI points and unimodal and Gaussians RRI and heart rate distribution graphics. As such, disturbing phenomena like the Valsalva manoeuvre were excluded. For frequency domain analysis, the best AR model order was chosen.

#### Stress questionnaires

If children and parents agreed to provide a saliva or hair sample for cortisol analysis, the children were individually interviewed by a trained researcher to obtain information about their life events *(Coddington Life Events Scale)*, daily hassles and uplifts *(Daily Hassles and Uplifts Scale)*, emotions *(Basic Emotions)* and coping strategy *(Coping Questionnaire)*. Furthermore, parents were asked to report on their child’s behavioral and emotional problems (*Strengths and Difficulties Questionnaire*). Only children from primary school were eligible to fill in the questionnaires (not kindergarten children).

##### Life events

The ‘Coddington Life Events Scale’ for children (CLES-C) [35] was used to identify potential physical and mental health problems arising from psychological causalities (reliability: r = 0.69; construct validity = 0.45). The English questionnaire was translated professionally into Dutch using a translation and back-translation process to ensure identical meaning. This validated 36-item questionnaire measured the frequency and timing of events in the last year relevant for this age group and resulted in a ‘life change units’ score for the time periods 0-3, 0-6, 0-9 and 0-12 months ago. To limit recall bias and to increase the accuracy of reporting in time, interviewers used calendar events such as birthdays, summer holidays and Easter [36]. Moreover, drawings and pictures were used to clarify some of the most difficult events, such as ‘juvenile court’, ‘respect’ etc. Children with a score above the age-specific cut-off were considered to be at higher risk to suffer from psychological problems. Apart from the total event score (both negative and neutral events), also a score for exclusively negative life events was calculated.

##### Daily hassles and uplifts

The children’s daily hassles (CHS) and daily uplifts (CUS) scales of Kanner et al. [37] contain 25 hassles and 25 uplifts, respectively (internal consistency: alpha = 0.87). Also for children as young as 5 and 6 years old, an internal consistency of 0.85 was shown and daily hassles correlated with parental reported behavioral problems [38]. Hassles refer to irritating, frustrating or distressing demands that characterize everyday transactions with the environment. Uplifts refer to positive experiences such as the joy derived from friendship, relief at hearing good news and so on. Children were asked to check which hassles and uplifts occurred during the last month. Furthermore, they were asked to rate whether they felt ‘not bad’, ‘sort of bad’, or ‘very bad’ as a result of the hassle and whether they felt ‘OK’, ‘sort of good’ or ‘very good’ as a result of the uplift. Both a total frequency, a frequency of higher intensity hassles and uplifts (‘sort of bad’ or ‘very bad’ and ‘sort of good’ or ‘very good’, respectively) and an intensity score can be calculated.

##### Emotions

Children were questioned about their recent feelings. As in the study of Zimmer-Gembeck [39], the feelings anger, anxiety, sadness and happiness were rated on a 0 to 10 Likert-scale (0 ‘not at all’ to 10 ‘very strong’). To help the children understand these distinct feelings, pictures of a social skills training game for very young children were displayed next to the question [40]. These basic emotions are understandable for infants and children [41] and can therefore uncomplicatedly be used in our population.

##### Coping

The children were asked what they usually do when they are confronted with problems or when they are upset by using an 8 item-questionnaire, with ‘never’, ‘sometimes’ or ‘often’ as response alternatives. This questionnaire was previously used in the CASE-study (Child and Adolescent Self-harm in Europe) [42] and translated into Dutch and substantially pilot-tested for a population of Belgian adolescents [43]. Although no psychometric data on this coping questionnaire was available for our age group, other coping questionnaires have been used with children’s self-report [44] and acceptable repeatability was shown in 5 to 6-year old children with open-ended questions (r between 0.67 and 0.77) [38]. The answers were classified as emotion- versus problem-focused coping, based on the transactional model of Lazarus and Folkman [45]. Emotion-focused coping is aimed at regulating emotional stress while problem-focused coping deals with the problem and makes changes in the disturbed and stress-inducing person-environment relationship.

##### Strengths and difficulties questionnaire

Parents were asked to complete the standardized ‘Strengths and Difficulties Questionnaire’ (SDQ) (reliability: ICC = 0.80; concurrent validity: r = 0.70) [46], reporting the emotional problems of their child over the past six months. For each of the 25 statements, parents could answer: ‘not true’ (0), ‘somewhat true’ (1) and ‘certainly true’ (2). The statements were divided in 5 subscales of 5 items each: emotional problems, conduct problems, hyperactivity-inattention behavior, peer problems, and prosocial behavior. Subscale scores were computed by summing scores on relevant items (after recoding reversed items). Higher scores on the prosocial behavior subscale reflect strengths, whereas higher scores on the other four subscales reflect difficulties [47].

#### Body composition measurements

##### Routine anthropometry

The routine anthropometric measurements were carried out by two trained observers at school to improve intra- and inter-observer reliability. Routine anthropometric measurements included measurement of the child’s weight, height, body mass index, leg-to-leg impedance, skin fold thicknesses (triceps and subscapular), circumference of mid-upper arm, hip, waist and neck and were performed in accordance with the standardized procedures of the IDEFICS project [17,48,49].

##### Fat mass determination by air displacement plethysmography (ADP)

To obtain reliable and valid body composition measurements, the ADP technique was conducted by the same person over all survey periods. This method is currently considered a good reference technique for body composition measurements with a quick, comfortable, automated, non-invasive and safe measurement process, making it feasible for children [50].

Body volume was measured by ADP (BODPOD®, Software version 4.2.4, Life Measurement Inc, Cranlea and Co, Birmingham, United Kingdom) using standardized procedures [51]. Children had to refrain from physical activity and food consumption two hours before the measurement. The device was calibrated daily according to the manufacturer's guidelines. Children were measured twice in tight-fitting bathing suit with swim cap to rule out air trapped in clothes and hair. Thoracic gas volume was predicted by the software with a validated child-specific equation [50] and fat mass percentage was calculated using the equation reported by Wells [52]. If the first and second measurement of the body volume differed more than 150 ml, a third measurement was performed.

#### Diet

##### Children’s Eating habits questionnaire (CEHQ)- food frequency questionnaire (FFQ) (parent-reported)

The CEHQ-FFQ investigates food consumption frequency and behaviors associated with obesity and general health in children. This 43 food-item-containing instrument was developed and validated within the IDEFICS project [53-55] and is used as a screening instrument to investigate dietary habits and food consumption frequency in children. Parents were asked to report on the frequency of their child’s consumption of selected food items in a typical week during the preceding 4 weeks, outside the school canteen or childcare meal provision settings, using the following response options: ‘never/less than once a week’, ‘1-3 times a week’, ‘4-6 times a week’, ‘1 time per day’, ‘2 times per day’, ‘3 times per day’, ‘4 or more times per day’ or ‘I have no idea’. Frequencies of intake were assessed without quantifying portion sizes.

##### Dutch eating behavior questionnaire (DEBQ) (child-reported)

In the DEBQ, a 33-item questionnaire (reliability: r = 0.87-0.90), three types of eating behavior can be identified in children: eating in response to negative emotions (emotional eating), eating in response to the sight or smell of food (external eating) and eating less than desired to lose or maintain body weight (restraint eating). In all three types of eating behavior, the appropriate self-regulating mechanism of food intake is diminished or lost. Children could answer the questions with ‘never’, ‘almost never’, ‘sometimes’, ‘often’ or ‘very often’ as response alternatives [56].

### Statistical methods

Population characteristics and participation and drop-out rates were statistically analyzed using PASW Statistics Program version 19.0.0 (SPSS Inc, IBM, IL, USA). Participation information was examined for the baseline (2010) and first follow-up (2011) survey period, for each examination module separately. Consent-percentage and drop-out proportion were calculated as the percentage of children with informed consent on the total number of eligible children and the percentage of children with informed consent who finally did not participate to the test, respectively. Information on family structure was dichotomized: a traditional family structure included children living with both biological parents; all other family types were categorized as non-traditional. Parental education was evaluated according to the ISCED classification (level 0 ‘pre-primary education’, 1 ‘primary education’, 2 ‘lower secondary education’, 3 ‘upper secondary education’, 4 ‘post-secondary non-tertiary education’, 5 ‘first stage of tertiary education’, 6 ‘second stage of tertiary education’) [57].

Sample size calculations, performed with the SAS System, indicated that a population of 500 children would allow to calculate correlation coefficients with 95% confidence within a 0.10 confidence interval of the true correlation coefficient of the population. This sample size additionally allows to observe a 10% increase in fat content with 95% confidence and 80% power. Also, this sample size allows to perform multiple regression analysis with BMI as dependent and stress as independent variable, adjusted for 6 covariates (i.e. child’s age, sex, socio-economic status, sleep, diet and physical activity) (N = 473) (SAS POWER procedure, Type III F Test in Multiple Regression).

### Future analytical plan

The feasibility and the interrelationship of the different stress measurements (questionnaires, cortisol in different biological samples, HRV) will be studied. This will be done using regression analyses corrected for age and sex, a hierarchical linear model for salivary cortisol (to model the diurnal pattern of cortisol) and a triad analysis allowing to compare more than two measurements at the same time (to examine which method may most accurately indicate true childhood stress) [58]. Secondly, regression models stratified for gender and corrected for age can verify the mutual relationships between stress and lifestyle related factors (sleep, diet and physical activity). Also, the possible mediation effect of psychological eating behavior in the stress-diet relationship will be tested. To reduce multicollinearity and complexity, principal component analysis will be executed on the stress questionnaires and lifestyle related factors. Finally, the association between stress and obesity will be tested both cross-sectionally and longitudinally (with repeated measures analysis). For this purpose, regression analyses will be executed correcting for the lifestyle related factors. Moreover, using mediation analyses the effect of lifestyle related factors on the stress versus body composition relationship will be examined. For a mediation effect, the relationship between the independent variable and the dependent variable should be significantly reduced after controlling for the mediator. This indirect effect will be formally tested with bootstrapping in SPSS [59] or in the more advanced Mplus software.

In all above-mentioned analyses, body composition parameters will be examined as continuous variables, such as body fat percentages, fat free mass percentages, BMI z-scores etc., instead of comparing obese versus non-obese groups.

## Results

### Participation rate

Table 1 presents, the consent- and drop-out-percentage, as well as the number of cases valid for data-analysis for each measurement module that took place at baseline and first follow-up. In the baseline and first follow-up survey, participation proportions of 68.7% (N = 523/761) and 65.8% (N = 418/635) were obtained, respectively. Willingness to participate from baseline to first follow-up (i.e. consent percentage) was 71.3% (N = 453/635).

**Table 1:** Participation numbers, separately for baseline survey (2010) and first follow-up (2011) of ChiBS in Aalter, Belgium

	**Baseline survey (2010)**						**First follow-up (2011)**					
	**Contacted N = 761**						**Contacted N = 635**^**a**^					
	**Eligible**	**Informed consent**	**Consent percentage**^**b**^	**Participated**	**Drop-out proportion**^**c**^	**Valid for analysis**^**d**^	**Eligible**	**Informed consent**	**Consent percentage**	**Participated**	**Drop-out proportion**	**Valid for analysis**
stress questionnaires	598^e^	563	94.2	523	7.1	523	635	453	71.3	418	7.7	418
salivary cortisol	598^e^	495	82.8	454	8.3	439	n/a	n/a	n/a	n/a	n/a	n/a
hair cortisol	293^f^	231	78.8	223	3.5	223	n/a	n/a	n/a	n/a	n/a	n/a
serum cortisol	474^g^	406	85.7	357	12.1	272^h^	n/a	n/a	n/a	n/a	n/a	n/a
HRV	761	513	67.4	475	7.4	460	635	453	71.3	437	3.5	412
ADP	761	513	67.4	497	3.1	497	635	453	71.3	453	0.0	453
weight and height	761	761	100	750	1.5	750	635	453	71.3	453	0.0	453

^a^ all children who participated to the BODPOD® measurements and/or stress questionnaires in 2010.

^b^ percentage of informed consents on the total number of eligible children.

^c^ percentage of children for who consent was given that did ultimately not participate (e.g. fear, test too difficult, defects of equipment or machinery).

^d^ number of children of whom data was valid for analysis after data-cleaning.

^e^ primary school children (boys and girls).

^f^ primary school girls.

^g^ limited to children from whom a salivary and/or hair sample was available.

^h^ as serum samples were also used to analyse other biological components, an insufficient amount of the sample for cortisol analyses was the largest limiting factor.

*ADP* air displacement plethysmography, *HRV* heart rate variability, n/a = not applicable as salivary, hair and serum sampling were not repeated in 2011.

### Socio-demographic characteristics

Table 2 shows the children’s and parental socio-demographic characteristics of participants with a baseline assessment of the stress questionnaires (ChiBS participants) compared to non-participating children in the ChiBS study.

**Table 2:** Socio-demographic characteristics of participating and non-participating children in the ChiBS study (2010, Aalter, Belgium)

	ChiBS participants*		ChiBS non-participants**		**χ²-test**
	**%**	**N**	**%**	**N**	**p-value**
**child characteristics**					
**sex**					0.634
male	49.3	258	51.3	117	
female	50.7	265	48.7	111	
**age (years)**					<0.001
5	0.4	2	55.7	127	
6	11.9	62	19.3	44	
7	24.7	129	7.5	17	
8	26.6	139	6.6	15	
9	24.9	130	7.0	16	
10	11.1	58	3.1	7	
11	0.6	3	0.9	2	
**BMI**					0.550
underweight	13.6	71	11.8	27	
normal	79.3	415	82.0	187	
overweight	5.5	29	5.7	13	
obese	1.5	8	0.4	1	
**parental characteristics**					
**household income** (missing = 108)			(missing n = 35)		0.377
low to low/medium	5.3	21	5.7	11	
medium	3.3	172	38.2	87	
high/medium	20.7	108	23.2	53	
high	21.5	112	18.4	42	
**education (ISCED)** (missing n = 30)			(missing n = 12)		0.163
level 1	0.6	3	0.9	2	
level 2	0.6	3	2.6	6	
level 3	24.7	129	27.6	63	
level 4	20.7	108	18.9	43	
level 5 or higher	47.8	250	44.7	102	
**family structure** (missing n = 32)			(missing n = 13)		0.066
traditional	76.9	402	82.5	188	
non-traditional	17.0	89	11.8	27	
**migrant status**					
father migrant	1.1	6	2.2	5	0.770
mother migrant	3.4	18	3.1	7	0.274

* children participating in the baseline ChiBS stress-questionnaire; ** children participating in the IDEFICS study but not in the ChiBS study; *BMI* body mass index with cut-offs as determined by the International Obesity Task Force classification; household income categories based on national statistics, *ISCED* International Standard Classification for EDucation (1 ‘primary education’, 2 ‘lower secondary education’, 3 ‘upper secondary education’, 4 ‘post-secondary non-tertiary education’, 5 or higher ‘tertiary education’); traditional family structure: children living with both biological parents – non-traditional family structure: all other family structures.

## Discussion

The ChiBS cohort provides valuable data and new insights into the influence of chronic psychosocial stress on changes in body composition, and its interaction with food intake regulation, physical activity/sedentary behavior and sleep in young children. In addition, this study allows an in-depth investigation of the validity of the different methods that were used to assess stress levels in children. A main challenge of the ChiBS study is to gather a large population of children willing to participate on a long-term, longitudinal basis. The large consent percentage from baseline to first follow-up (N = 453/635; 71.3%) was already promising. Efforts are done to raise the participation rate in the second measurement as well, as attrition is known to increase over the years in longitudinal studies (e.g. annual feed-back to the parents, little incentive for the children at participation, study-information by various channels such as letters by post, website and telephone calls).

### Sampling and representativeness

In view of a number of budgetary and logistical advantages, it was decided to embed this study within the European IDEFICS project, as already mentioned above. However, this approach inherently implied the introduction of a selection bias, i.e. parents interested and willing to participate in IDEFICS may also be more motivated to participate in another study, e.g. ChiBS. The high consent percentages of the baseline ChiBS examination modules (ranging from 67% to more than 90%) must be interpreted in this context. Even though it should be noted that all studies based on the recruitment of volunteers to participate may be subjected to this type of selection bias. On the other hand, embedding ChiBS within IDEFICS, which is already burdening children and parents, may have decreased participation to the additional ChiBS project.

However, we did not find significant socio-demographic differences between ChiBS participants and ChiBS non-participants (Table 2), indicating that there was no further participation bias introduced by the subjects included in the ChiBS study in comparison with the existing IDEFICS cohort. Table 2 however shows that ChiBS non-participants were younger than ChiBS participants. The differential attrition for 5-year olds (as indicated in Table 2) can be explained by the introduction of eligibility criteria: an age cut-off for stress measurements (i.e. first year of elementary school) was introduced because of reliability issues with kindergarten children. As we recruited the children at classroom-level and not at individual level, the participation of 6 year olds may also be artificially distorted as some children of the last kindergarten-year may already be 6 years old and were therefore not included in this study. The goal was thus to cover all primary school ages, by examining the first to fourth class-year (commonly covering the ages between 6 and 10) at baseline and by examining third to last year of primary school (normally covering the ages between 8 and 12) at the final follow-up. This way, all primary school years are covered by the end of this study.

To further examine the representativeness of our study population, we compared the study sample with socio-demographic characteristics of the general population in Flanders. This socio-demographic information was obtained through consulting statistics of the Flemish authorities and ‘Child and Family’ [60-62] and resulted in the following findings. Parents of the children participating to ChiBS are higher educated and less often of migrant origin compared to the general Flemish population (47.8% versus 31.7% of ISCED 5 or higher; 3.4% versus 6.4% of migrants; respectively). Additionally, a traditional two-parent family structure is more prevalent in ChiBS participants compared to the general Flemish population (76.9% versus 66.1% of traditional family structures respectively), indicating that the ChiBS children belong to a higher socio-economic class compared to the general Flemish population. It should however be noted that these findings are inherently related to the overall higher socio-demographic characteristics of the city of Aalter compared to the general Flemish population [63]. The overweight and obesity percentages observed in the baseline ChiBS survey are low compared to the most recent reference data available for Flemish children. The Flemish Growth Survey, conducted between 2002-2004 in 16000 Flemish children and adolescents, demonstrated overweight and obesity percentages of 14.2% and 3% for female participants respectively, and 11.8% and 2.6% of overweight and obesity for male participants respectively (2-18 years old) [64]. We acknowledge our low percentages as a study limitation. Nevertheless, our study population still allows to examine the evolution and changes in physical fitness and body composition parameters (such as fat percentage, fat free mass) as continuous variables longitudinally, although generalization of results to high overweight populations or studying differences between obese and non-obese children will not be possible. Moreover, we are applying for additional funding to prolong the follow-up phase to the ages of adolescence as this would provide sensitive information on stress-related changes from childhood to adolescence.

### Participation and drop-out proportions

To increase willingness for participation, we decided to split the informed consent such that the participants could refuse participation to single examination modules. Nevertheless, this has led to different participation rates for each examination module and a low number of children participating to the whole ChiBS examination battery. Consequently, this implies studying sub-groups of ChiBS participants for investigating separate research questions and a limited number of children in whom the whole ChiBS model is examined.

Remarkably higher participation rates were recorded for blood withdrawal, saliva sampling and hair sampling compared to HRV and ADP measurements. This may be due to the extra effort required from the parents to visit the sports park. In contrast, blood sampling had the highest drop-out proportion because of the higher frequency of non-successful assessments (12.1%). Drop-out proportions for the other examination modules are explained as follows: some children failed to successfully complete the stress-questionnaires (7.1%), 8.3% of the parents refused or failed to take salivary samples, 3.5% of the girls had an insufficient length of hair (<6 cm) to perform the hair sampling, 7.4% of the HRV measurements were lost because of not keeping one’s appointment, children being too nervous for HRV registration or a temporarily technical failure of the HRV registration, 3.1% of the children did not show up for the ADP measurement or were not willing to sit in the BODPOD® instrument.

Nevertheless, we experienced all examination modules as feasible techniques in working with young children.

## Conclusions

The ChiBS project is unique in its setting and scope. Its standardized and longitudinal approach provides valuable data and new insights into the relationship between stress and changes in body composition in young children. In addition, this study allows an in-depth investigation of the validity of the different methods that were used to assess stress levels in children.

## Competing interests

The authors declare that they have no competing interests.

## Authors’ contributions

All authors have made a substantial contribution to this manuscript based on the three conditions: study design, manuscript editing and final approval. NM and BV were the major responsible persons for the practical organization and data collection, statistical analyses, literature research and wrote the first manuscript draft. Furthermore, KV was also a major responsible person for the IDEFICS data collection. All authors read and approved the final manuscript.

## Authors’ information

Joint first authorship: Nathalie Michels and Barbara Vanaelst.

## Supplementary Material

Additional file 1 Manual for saliva sampling. Click here for file
