# Supplementary material for: Children’s Body composition and Stress – the ChiBS study: aims, design, methods, population and participation characteristics
Source: Arch Public Health. 2012 Aug 9;70(1):17. doi: 10.1186/0778-7367-70-17 (PMC3524083; doi:10.1186/0778-7367-70-17)
Supplement: Additional file 1 — Manual for saliva sampling. [file 0778-7367-70-17-S1.pdf]

UNIVERSITEIT GENT

Instructions saliva collection

The purpose of this saliva collection is to measure stress by analyzing the hormone cortisol in saliva. Apart from the instructions with 2 checklists, you will also have 2 bags each containing 4 cotton swabs. With these swabs you can collect saliva from your child.

Time points for collection (the time points have to be followed strictly) during these 2 days:

On ____________________ and __________________ (dates completed by the researcher) (if you want to chose another date, please contact the researcher on the phone number below!)

1. **Immediately after awakening, just after opening the eyes when the child is still in bed!**
2. **30 minutes after awakening**
3. **60 minutes after awakening**
4. **In the evening: at least one hour after dinner but between 7 and 8 PM**

Please pay attention to the following guidelines as it is of vital importance to standardize the sampling:

1) Be **punctual** in following the time points. In order to sample as quick as possible after awakening, leave the bag, a ballpoint and a watch beside your bed. Please, take a good look at the sampling steps the day before and familiarize yourself with the saliva tubes.
2) During **1 hour before sampling** your child should not:
   -**eat or drink** (except water)
   -**brush its teeth**
   During **2 hours before sampling** your child should not:
   -do intensive **physical activity**
   This means that your child should stay fasting until 1 hour after awakening and that teeth brushing should be postponed until then. Please, plan your morning routine by giving breakfast just before leaving to school or try to get up a bit earlier.
3) Pay attention to it that your child takes **as less medication as possible** on the sampling day and does **not consume caffeine** (coffee, cola, some energy drinks,…).
4) If you have more than 1 child who is sampling, be sure not to switch the samples.

Please, follow these steps for sampling:

1) Make sure you have some time free during the above-mentioned time points.
2) Take 1 bag per day and 1 tube per moment. On the tube the ID-number of your child is written with after the line a number between 1 and 8. These numbers should be used **chronological**. On day 1 you use tube 1 to 4, on day 2 you use tube 5 to 8. E.g. 1410000/1 immediately after awakening, 1410000/2 30 minutes after awakening,…
3) Open the tube and take the cotton swab. The small tube inside is for the analysis, please don’t take it away.

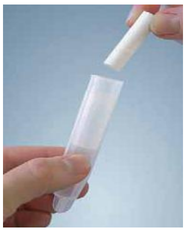

4) Your child has to take **the whole cotton swab in the mouth** and move it around inside the mouth during 2 minutes, until the cotton roll is completely saturated with saliva. It is very important to collect as much saliva as possible! **Don't let your child chew or suck on it.**
5) Place the cotton roll back in the tube and **close the tube tightly**!
6) **Write the exact time point, the date and name of your child on the tube (with ballpoint). Don't forget to answer the questions in the checklist!**
7) Place the tube back in the bag.
8) Place the used tube immediately in the **refrigerator** (not on room temperature!).
9) Please bring the 8 tubes and the 2 checklists back to school on ______________________ (date completed by the researcher). The researchers will collect the tubes during the first morning session.

Please contact the researcher in the following situations:

- If you have forgotten to bring the tubes back to school on the instructed date.
- If you could not follow the guidelines on the instructed date (e.g. the child was sick, the time points could not be followed). We will suggest you another day so that we will be able to collect the tubes on the next day.
- If you have some problems or questions.

Thank you in advance for your helpful cooperation!
